# A penalized integrative deep neural network for variable selection among multiple omics datasets

**DOI:** 10.1002/qub2.51

**Published:** 2024-06-07

**Authors:** Yang Li, Xiaonan Ren, Haochen Yu, Tao Sun, Shuangge Ma

**Affiliations:** ^1^ Center for Applied Statistics School of Statistics Renmin University of China Beijing China; ^2^ Department of Biostatistics Yale University New Haven Connecticut USA

**Keywords:** deep learning, integrative analysis, multiple omics datasets, variable selection

## Abstract

Deep learning has been increasingly popular in omics data analysis. Recent works incorporating variable selection into deep learning have greatly enhanced the model’s interpretability. However, because deep learning desires a large sample size, the existing methods may result in uncertain findings when the dataset has a small sample size, commonly seen in omics data analysis. With the explosion and availability of omics data from multiple populations/studies, the existing methods naively pool them into one dataset to enhance the sample size while ignoring that variable structures can differ across datasets, which might lead to inaccurate variable selection results. We propose a penalized integrative deep neural network (PIN) to simultaneously select important variables from multiple datasets. PIN directly aggregates multiple datasets as input and considers both homogeneity and heterogeneity situations among multiple datasets in an integrative analysis framework. Results from extensive simulation studies and applications of PIN to gene expression datasets from elders with different cognitive statuses or ovarian cancer patients at different stages demonstrate that PIN outperforms existing methods with considerably improved performance among multiple datasets. The source code is freely available on Github (rucliyang/PINFunc). We speculate that the proposed PIN method will promote the identification of disease‐related important variables based on multiple studies/datasets from diverse origins.

## 1 INTRODUCTION

Recent advances in high‐throughput technologies have generated large volumes of omics data. The high dimensionality and complex structure in omics data have posed great challenges for statistical methods [1, 2]. Deep learning has made extraordinary achievements in precision medicine by effectively extracting information from the complex omics data [3, 4].

One major task in deep learning is to enhance its interpretability [5, 6]. In particular, it is crucial to identify important input variables in the neural network. Several deep feature selection (DFS) methods [7, 8, 9] have been proposed by incorporating variable selection penalties into the neural network model. However, the small sample size in one single dataset, common in omics studies, could lead to uncertain and unstable findings using traditional deep learning methods [10, 11, 12]. Recently, the explosion and availability of omics data from multiple populations/studies have greatly boosted the sample size for one specific research aim, such as cognitive studies with each dataset containing gene expression data from elders at different cognitive stages and ovarian cancer studies with each study recruiting patients at different cancer subtypes. One could naively apply the DFS methods by pooling multiple datasets into one dataset before analysis. However, the pooling procedure assumes that different datasets share common variable structures, which may not be true in real applications, and therefore might produce inaccurate variable selection results [13, 14].

**FIGURE 1:**
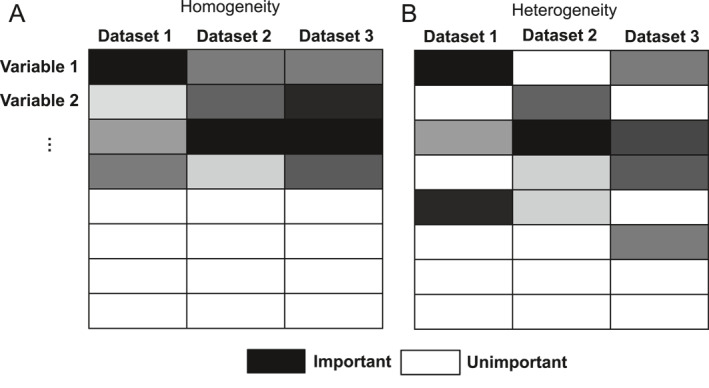
Schematic diagrams of important and unimportant variables in multiple datasets under (A) homogeneity and (B) heterogeneity situations. The columns represent the different datasets, and the rows indicate the individual variables. A black block denotes that the corresponding variable is important. The shade of the color shows magnitudes of variable effects.

To address the limitations of DFS methods in analyzing multiple omics datasets, we consider the integrative analysis framework for variable selection [14, 15, 16, 17, 18, 19]. The integrative analysis is a multi‐dataset‐based variable selection approach that is more accurate and stable than the conventional variable selection approaches in analyzing multiple omics datasets [18, 19]. One significant advantage of integrative analysis is that it enables simultaneous raw data aggregation and variable selection among multiple datasets. Specifically, integrative analysis treats each variable across all datasets as a single group and uses regularization techniques to identify important groupings. Moreover, integrative analysis naturally incorporates the homogeneity situation where important variables are generally consistent across datasets (Figure 1A) and the heterogeneity situation where important variables overlap but are different across datasets (Figure 1B). In particular, the magnitudes and directions of variable effects could vary across datasets in integrative analysis, which renders great flexibility in real applications. However, the existing integrative analysis approaches usually assume a linear relationship between variables and outcomes, ignoring the complex variable structures in high‐dimensional omics data.

By synergizing deep learning and integrative analysis, we propose a novel penalized integrative deep neural network (PIN) for variable selection among multiple omics datasets. Specifically, we first construct a deep neural network that effectively approximates the complex variable structures in each dataset. Then, we select important variables by performing integrative analysis on these neural networks from all datasets. Moreover, we develop two types of PIN to address the homogeneity and heterogeneity situations: the homogeneity penalized integrative deep neural network (HoPIN) and the heterogeneity penalized integrative deep neural network (HePIN). A data‐driven selection procedure for choosing between HoPIN and HePIN is illustrated in real data applications. The Supporting Materials provides two toy examples demonstrating how to implement PIN step‐by‐step. In the following sections, we will describe the proposed method and evaluate its variable selection and prediction performance using simulation studies, three Alzheimer’s disease (AD) datasets, and four ovarian cancer datasets.

## 2 RESULTS

### 2.1 Overview of PIN

**FIGURE 2:**
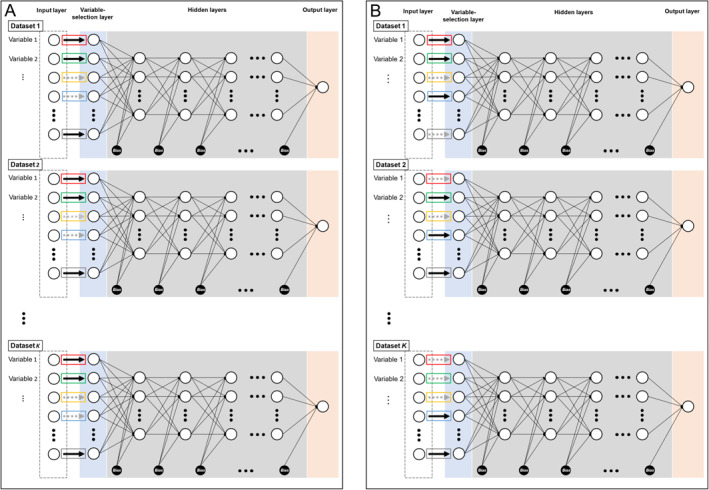
The network topology of (A) HoPIN and (B) HePIN. Each neural network corresponds to one dataset and contains a variable‐selection layer, whose nodes connect with the input layer nodes in a one‐to‐one fashion. The arrow inside each box denotes the weight of the connection between the input layer node and the variable‐selection layer node. The solid or dashed arrow indicates whether the input variable is important (solid) or not (dashed). Take Variable 1 as an example. For HoPIN in (A), weights in the red boxes across all the neural networks are regarded as one group for Variable 1 (i.e., ***w***_1_). The selection of Variable 1 among datasets is achieved by applying a group penalty to the weight group ***w***_1_. For HePIN in (B), as Variable 1 is only important in dataset 1 and unimportant in the rest datasets, the selection of Variable 1 among datasets can be achieved by applying a sparse group penalty to the weight group ***w***_1_. HePIN, heterogeneity penalized integrative deep neural network; HoPIN, homogeneity penalized integrative deep neural network.

PIN is a neural‐network‐based integrative analysis framework that enables simultaneous selection of important variables from multiple datasets with high‐dimensional variables. The architectures of PIN are illustrated in Figure 2. Take the architecture in Figure 2A as an example. Each dataset corresponds to one DFS network. In contrast to a conventional deep neural network, the DFS network implements a one‐to‐one connection layer between the input and the first hidden layers. The weights between the input and connection layers represent the variable importance levels. For each variable, weights from all datasets constitute one single group. The PIN achieves multi‐dataset variable selection by applying a group or sparse‐group penalty on the weights groups (see Methods).

We propose two PIN methods: the homogeneity and heterogeneity penalized integrative deep neural networks (HoPIN and HePIN) for homogeneity and heterogeneity situations. The network topologies of HoPIN and HePIN are demonstrated in Figure 2. The difference between HoPIN and HePIN lies in the one‐to‐one connection between the input and the first hidden layer. Specifically, for HoPIN, we impose the group lasso on groupings of weights across neural networks ***w***_*j*_, j = 1, 2, …, *p*, indicating that variables within the same group are either all‐important or all‐unimportant. For HePIN, we impose the sparse group lasso on groupings of weights, indicating that some of the variables within one group can be important while the rest can be unimportant. Our PIN methods have several advantages over existing single‐dataset‐based DFS methods: (1) PIN can efficiently overcome the instability and uncertainty issue in the single‐dataset DFS methods. (2) PIN can handle both homogeneity and heterogeneity situations and thus has a broad range of applications. Two toy examples illustrating how to implement PIN under homogeneity and heterogeneity situations are illustrated in the Supporting Materials.

### 2.2 Real data analysis on Alzheimer’s disease datasets

Three datasets from the Alzheimer’s Disease Neuroimaging Initiative (ADNI) are analyzed: the cognitive normal (CN) dataset (244 elders), the mild cognitive impairment (MCI) dataset (377 elders), and the AD dataset (113 elders). Our goal is to find gene expression markers associated with cognitive decline in elders. We use the AD assessment scale 13 (ADAS13) as the cognitive evaluation metric, with a larger value indicating a more severe cognitive status. The gene expression data are generated from the same genotyping platform across the three datasets.

AD is a chronic disease that gradually progresses from CN to MCI to dementia. It is well recognized that AD‐related brain pathology occurs before the clinical sign of cognitive decline appears [20]. Therefore, patients at different cognitive stages may share a common list of important markers.

**FIGURE 3:**
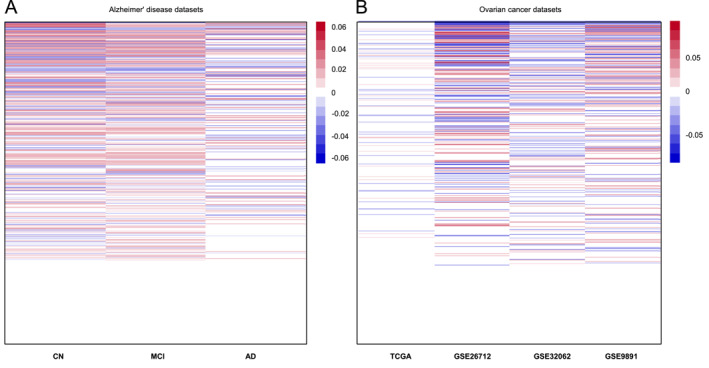
(A) Gene expression effects on Alzheimer’s disease assessment scale 13 (ADAS13) in three Alzheimer’s disease datasets. (B) Gene expression effects on survival time in four ovarian cancer datasets. The columns represent the different datasets, and the rows indicate the individual genes. Positive effect, negative effect, and magnitude of gene effects are shown by red, blue, and the shade of the color. AD, Alzheimer’s disease; CN, cognitive normal; MCI, mild cognitive impairment.

We propose a data‐driven graphical selection procedure to choose between HoPIN and HePIN. Specifically, we use linear regressions to estimate the effect of each gene on ADAS13 in each dataset. As shown in Figure 3A, the important genes are generally consistent across the three datasets, suggesting a homogeneity situation in this application. The effects’ directions (in colors) and magnitudes (in shades) vary for each gene across datasets. As a result, we analyze the ADNI datasets using the proposed HoPIN and compare its performance with HoInte (homogeneity penalized integrative analysis), p‐DFS (pooled DFS), and s‐DFS (separate DFS).

Before analysis, we normalize the gene expression data following Liu et al. [16] and select the top 500 genes associated with ADAS13 ranked by *p*‐values. We evaluate the variable selection performance using the observed occurrence index (OOI) [21] and the prediction performance using the mean of squared errors (MSE). Specifically, we randomly select 80% subjects as training data and the other 20% as test data. We train the model in the training data and obtain MSE in the test data. As the true set of important variables is unknown, we evaluate the variable selection performance using the observed occurrence index (OOI) [21]. To be specific, we first perform variable selection in the training data and obtain a list of important variables. Then, we conduct bootstrap and perform variable selection in the bootstrap dataset. The bootstrap process is repeated 50 times. We calculate the proportion of times each variable identified in the original training data is selected out of 50 bootstrap replications and calculate the average across all variables as the OOI value. A higher OOI value corresponds to better variable selection stability. A smaller SSE value indicates a better prediction accuracy.

We evaluate four methods using the ADNI datasets: (1) the traditional homogeneity integrative analysis method [15], which assumes linear variable effects (denote as ‘HoInte’); (2) the traditional DFS approach [8] being applied to each separate dataset (denote as ‘s‐DFS’); (3) the pooled‐dataset DFS approach, which pools multiple datasets into one and applies the traditional DFS method (denote as ‘p‐DFS’); and (4) the proposed HoPIN method.

For HoPIN, the best performing model uses the following parameters: the number of hidden layers = 2, learning rate = 0.3, penalty parameter for the variable selection layer *λ* = 0.02, and penalty parameter for preventing overfitting *α* = 0.00001. HoPIN discovers 22 important genes (Table S1 in the Supporting Materials). For variable selection stability, the OOI value for HoPIN is 0.58, greater than HoInte (0.44), p‐DFS (0.45), and s‐DFS (0.43). Therefore, HoPIN is more stable than the rest of the methods in variable selection. For prediction accuracy in the test data, HoPIN gives an MSE value of 0.026, smaller than or similar to HoInte (0.027), p‐DFS (0.031), and s‐DFS (0.026). Overall, HoPIN achieves excellent selection stability and prediction accuracy in identifying important markers associated with cognitive decline in elders.

Among the important genes discovered by HoPIN, *SLC45A1* is widely known to be associated with intellectual disorders [22, 23]. *SAV1* is the core kinase for the Hippo signaling pathway regulating cell proliferation and apoptosis [24]. *EIF4EBP1* is associated with the regulation of neuroblasts, which are embryonic cells from which nerve fibers originate [25]. *PLBD2* is associated with the neuron renewal process [26]. These findings may provide valuable insights into the cognitive decline process among elders.

### 2.3 Real data analysis on ovarian cancer datasets

We use four datasets of ovarian cancer patients with complete survival time from the CuratedOvarianData R package [27]: TCGA dataset (290 subjects), GSE26712 dataset (129 subjects), GSE32062 dataset (121 subjects), and GSE9891 dataset (113 subjects). The four datasets are different from certain perspectives. Specifically, the four studies include patients from various regions and focus on different ovarian cancer types. We aim to find gene expression markers associated with ovarian cancer patient survival.

We use the data‐driven graphical selection procedure to choose between HoPIN and HePIN, like the AD data analysis. As depicted in Figure 3B, the important genes overlap but generally exhibit different patterns across datasets, indicating a heterogeneity situation in this application. Therefore, we use HePIN to investigate the heterogeneity of gene effects on ovarian cancer patient survival among the four datasets and compare its performance with HeInte (heterogeneity penalized integrative analysis) and s‐DFS.

We evaluate three methods using the TCGA datasets: (1) the traditional heterogeneity integrative analysis method [17], which assumes linear variable effects (denote as ‘HeInte’); (2) the traditional DFS approach being applied to each separate dataset (denote as ‘s‐DFS’); and (3) the proposed HePIN method.

We screened the top 500 genes associated with ovarian cancer patient survival. For HePIN, the best performing model has the following parameters: the number of hidden layers = 2, learning rate = 0.2, penalty parameters for variable selection *λ*_1_ = 0.05, *λ*_2_ = 0.1, and penalty parameter for preventing overfitting *α* = 0.00001. HePIN identifies 195 important genes (Table S2 in the Supporting Materials). For selection stability, the average OOI value of HePIN across the four datasets is 0.60, higher than HeInte (0.36) and s‐DFS (0.47). Therefore, HePIN achieves better variable selection stability than HeInte and s‐DFS. Moreover, HePIN (MSE 0.030) and s‐DFS (MSE 0.030) produce better prediction accuracy than HeInte (MSE 0.038) in the test data. Overall, the HePIN method successfully identifies important genes unique to each ovarian cancer dataset and achieves better selection stability and prediction accuracy than the existing methods.

Among the important genes shared in all four datasets selected by HePIN, *PDHB* is shown to have effects on the malignant proliferation of cancer cells [28, 29]. *NFS1* is shown to be associated with the programmed death of cancer cells [30]. *GKN1* is reported as an important marker in screening ovarian cancer patients [31]. In addition, *MICAL*, an important gene identified in TCGA, is a critical regulator of the epithelial–mesenchymal transition in ovarian cancer cells [32]. Another gene, *B4GALT5*, is found to be associated with survival in ovarian cancer patients [33]. These findings may provide valuable insights into gene expression effects on ovarian cancer in multiple cohorts.

### 2.4 Simulation results

We evaluate the performance of the PIN methods for variable selection under both homogeneity and heterogeneity situations. We consider both linear and nonlinear variable structures. The directions and magnitudes of effects for the same variable varied across different datasets. More details about the simulation settings are in the Methods Section.

For the homogeneity situation, we generate three datasets with 100 variables, in which variables 1–5 are important. We set the correlations between variables *i* and *j* as 0.5^|*i* − *j*|^. The four homogeneity methods (HoInte, s‐DFS, p‐DFS, and the proposed HoPIN method) are being evaluated on simulated homogeneity datasets. We evaluate model variable selection performance using four metrics: sensitivity (SEN), specificity (SPE), the geometric mean of sensitivity and specificity (GM), and the correct classification rate (CCR). The greater these metrics, the more capable the model is in variable selection. The prediction performance is measured using the MSE in independent test data. Specifically, we randomly select 80% subjects as training data and the rest as test data. The training data are used to train the model and select important variables, and the test data are used to obtain the MSE. We repeat this process 50 times.

**FIGURE 4:**
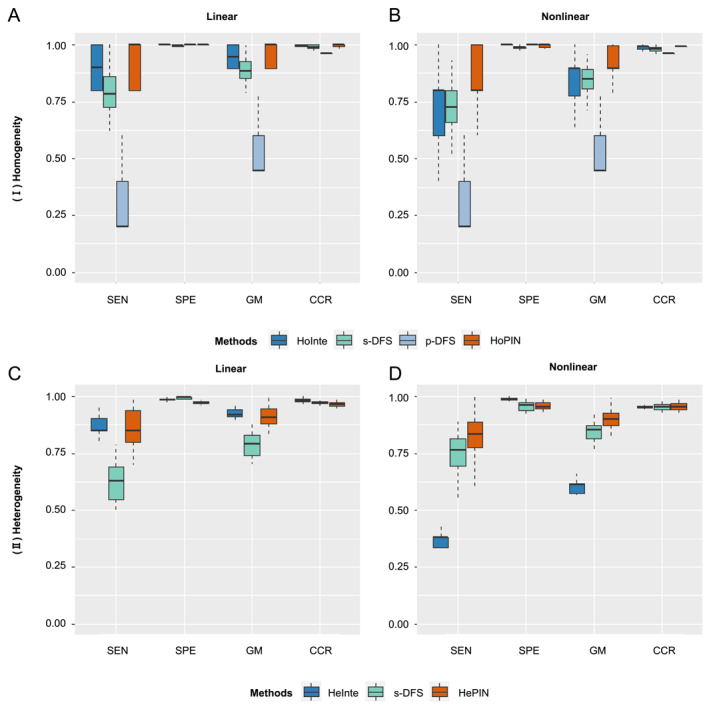
Boxplots of variable selection metrics (SEN, SPE, GM, and CCR) under the homogeneity (I) and heterogeneity (II) situations where the variable structures are linear or nonlinear. Three datasets are generated with a total of 525 individuals and 100 variables. For the homogeneity situation, we compare HoPIN with HoInte (the traditional homogeneity integrative analysis method under linear variable effects assumption), s‐DFS (applying the traditional deep features election method to each separate dataset and pooling the results together), and p‐DFS (pooling multiple datasets into one and applying the traditional DFS method). For the heterogeneity situation, we compare HePIN with HeInte (the traditional heterogeneity integrative analysis method under linear variable effects assumption) and s‐DFS (applying the traditional DFS method to each separate dataset and pooling the results together). Fifty replications of simulations are performed. SEN stands for sensitivity, SPE for specificity, GM for the geometric mean of sensitivity and specificity, and CCR for correct classification rate. Higher values of the four metrics indicate better selection performance. CCR, correct classification rate; GM, geometric mean; HePIN, heterogeneity penalized integrative deep neural network; HoPIN, homogeneity penalized integrative deep neural network; s‐DFS, separate DFS; SEN, sensitivity; SPE, specificity.

When the variable structures are linear, as illustrated in Figure 4A, HoPIN and HoInte generally achieve the most optimal variable selection performance based on all four metrics. For s‐DFS, its variable selection performance is less optimal than HoPIN because of each dataset’s relatively small sample size. For p‐DFS, its variable selection performance is the least optimal among all methods because it fails to handle situations where the directions of covariate effects are opposite across the simulated datasets. In particular, the naive pooling procedure used by p‐DFS may cancel out some non‐zero variable effects, making it hard to identify true important and unimportant variables. When the variable structures are nonlinear, HoPIN demonstrates almost the best variable selection capacity (Figure 4B). HoInte results in low accuracy because of its linearity assumption. The performance of s‐DFS is less optimal than HoPIN because of the limited sample size in each dataset. The p‐DFS method exhibits the lowest performance because it ignores the distinct variable structures among datasets and builds only one single neural network based on the pooled dataset, which may fail to select variables in individual datasets effectively. For prediction performance (Table 1(I)), when the variable structure is linear, HoInte and HoPIN produce smaller MSE values than p‐DFS and s‐DFS. When the variable structure is nonlinear, the four methods exhibit similar performance.

**TABLE 1:** The mean squared error averages and standard deviations under the homogeneity (I) and heterogeneity (II) situations when the variable structure is linear or nonlinear.

		Linear	Nonlinear
(I) Homogeneity	HoPIN	0.012 (0.002)	0.024 (0.004)
	HoInte	0.011 (0.002)	0.024 (0.004)
	p‐DFS	0.017 (0.004)	0.022 (0.005)
	s‐DFS	0.014 (0.003)	0.026 (0.005)
(II) Heterogeneity	HePIN	0.015 (0.003)	0.023 (0.004)
	HeInte	0.014 (0.002)	0.029 (0.004)
	s‐DFS	0.018 (0.004)	0.025 (0.005)

*Note*: The number of predictors is set at *p* = 100.

Abbreviations: HoPIN, homogeneity penalized integrative deep neural network; HoInte, homogeneity penalized integrative analysis; p‐DFS, pooled DFS; s‐DFS, separate DFS; HePIN, heterogeneity penalized integrative deep neural network; HeInte, heterogeneity penalized integrative analysis.

For the heterogeneity situation, we assume that the important variables in each dataset are overlapping but not identical. We simulate three datasets with 100 variables, and correlations between variables *i* and *j* as 0.5^|*i* − *j*|^. The evaluation criteria are the same as those used for the homogeneity situation. We compare our proposed HePIN to two alternative approaches: HeInte and s‐DFS. We conducted the simulations 50 times.

As shown in Figure 4(II), HeInte results in low accuracy in variable selection when the variable structure is nonlinear. Like in the homogeneity situation, the performance of s‐DFS is restricted because of the limited sample size in each dataset. The proposed HePIN generally outperforms HeInte and s‐DFS under linear and nonlinear settings. For prediction performance under the heterogeneity situation, as shown in Table 1(II), HeInte and HePIN produce smaller MSE values than s‐DFS when the variable structures are linear. When the variable structures are nonlinear, HePIN outperforms the rest methods.

We conduct additional simulations when the number of predictors is set at *p* = 500 for each dataset. The variable selection results are presented in Figure S1, and the prediction performance results are summarized in Table S3 in the Supporting Materials. Overall, the observations are consistent with the previous findings.

Furthermore, we conducted an additional simulation when there is no overlap of important variables among all datasets. The data settings are presented in the Supporting Materials, and variable selection results are presented in Figure S2. The proposed method HePIN performs well and is comparable to performing DFS separately for each dataset. In terms of prediction performance, HePIN outperforms s‐DFS due to the larger sample size.

## 3 DISCUSSION

We propose a novel penalized integrative deep neural network, PIN, for variable selection among multiple datasets. The proposed method selects important variables in multiple omics datasets by effectively modeling and integrating nonlinear variable structures in each dataset. Extensive simulations and real data applications demonstrate PIN’s satisfactory variable selection capacity and stability. Our method will promote the identification of disease‐related important variables based on multiple studies/datasets from diverse origins.

One practical issue in applying PIN is to decide whether the multi‐dataset complies with the homogeneity or heterogeneity situation. Existing integrative analysis literature made this decision based on subjective conjecture on how ‘similar’ or ‘different’ the datasets are [16, 17]. However, it is desirable to have explicit guidelines in real applications. Therefore, we propose a data‐driven graphical selection procedure described in two real applications. As depicted in Figure 3, the procedure helps distinguish homogeneity or heterogeneity among multiple datasets. In real data analysis, we prioritize the use of HePIN when the true scenario is unknown. On one hand, HePIN is more flexible than HoPIN, and HePIN includes the HoPIN model as a special case. An example is provided in the Supporting Materials to illustrate HePIN can also perform well under the homogeneity situation (Figure S3). On the other hand, given that heterogeneity always exists in the real world, using HePIN is a more cautious approach. In addition, two toy examples are provided in the Supporting Materials to illustrate the PIN implementation in detail under both homogeneity and heterogeneity situations.

The proposed method can be improved in multiple future directions. First, the interpretability of PIN can be further enhanced. In particular, the biological pathways via which the important genes influence clinical outcomes are not explicitly known. One potential solution is to implement gene regulatory pathways into the neural networks of PIN. Second, the proposed method currently handles only complete and continuous outcomes. In particular, we remove censored observations in analyzing the ovarian cancer survival data, resulting in possible information loss. Therefore, it is worthwhile extending the method to handle censored outcomes [34, 35] and other types such as categorical outcomes [36].

The proposed PIN is applied to extensive simulations, three AD datasets, and four ovarian cancer datasets to demonstrate its variable selection ability in multiple datasets with high‐dimensional variables. The proposed method stands as a novel integrative framework for biological discovery with the explosion and availability of multiple omics datasets from various sources.

## 4 CONCLUSION

To mitigate the uncertainty stemming from conventional deep learning approaches when applied to limited‐sample omics datasets, we strive to make full omics data from multiple populations/studies. We propose a new deep learning‐based integrative‐analysis variable selection method named PIN. The new method effectively selects important variables by capturing the complex nonlinear relationships between variables and by integrating information from multiple datasets. PIN can well handle different realistic situations (i.e., homogeneity and heterogeneity) in analyzing multiple datasets.

## 5 MATERIALS AND METHODS

### 5.1 Datasets

AD datasets: AD is a chronic disease that gradually progresses from the CN to MCI. Eventually, the disease progresses to AD with severe cognitive loss. Our AD datasets are obtained from the ADNI study, containing 734 individuals with gene expression data. The outcome of interest is the ADAS13, which assesses the severity of cognitive dysfunction. A greater value of ADAS13 indicates worse cognitive performance. The 734 individuals include three sub‐datasets: CN (244 individuals), MCI (377 individuals), and AD (113 individuals) [37].

Ovarian cancer datasets: The ovarian cancer datasets are obtained from the Bioconductor package “curatedOvarianData” [27]. We use four studies, including TCGA, GSE26712, GSE32062, and GSE9891. We are interested in identifying markers associated with the survival time of patients with ovarian cancer. We consider subjects whose survival time data were not missing. The sample sizes are 290, 129, 121, and 113 in TCGA, GSE26712, GSE32062, and GSE9891. The four datasets are quite different from multiple perspectives. First, the four datasets represent patients from different regions or countries. Specifically, TCGA, GSE26712, GSE32062, and GSE9891 recruit patients from the Washington University School of Medicine, the Memorial Sloan‐Kettering Cancer Center, Japan, and the Australian Ovarian Cancer Study, respectively. Second, the four datasets target different stages of ovarian cancer. For example, TCGA recruits high‐grade serous ovarian cancer (HGS‐OvCa) patients at stages II–IV; GSE26712 and GSE32062 include patients at stages III–IV; and GSE9891 evaluates patients with endometrioid invasive ovarian, fallopian tube, and peritoneal cancers.

### 5.2 Methods

#### 5.2.1 Notation, network architecture, and loss function

Assume we have *K* number of datasets, each containing *N*_*k*_ subjects with *k* = 1, 2, …, *K*. Denote $Y_{i}^{k}$ as the continuous response variable and $X_{i}^{k}$ as the *p*‐dimensional variable vector for the *i*th subject in the *k*th dataset. We assume $Y_{i}^{k}=f^{k}\;\left(X_{i}^{k}\;;\theta_{k}\right)+\epsilon_{i}^{k}$ , where *f* ^*k*^(∙) is an unknown function and ***θ***_*k*_ represents the parameter set for the *k*th dataset. Since *f* ^*k*^(∙) may take any nonlinear form, it is difficult to select important variables in the complex structure of *f* ^*k*^(∙). Moreover, it becomes even more complicated to perform variable selection across multiple datasets simultaneously.

We propose a PIN that can simultaneously model the unknown function *f* ^*k*^(∙) and perform variable selection across datasets. Specifically, for each dataset *k*, we approximate $f^{k}\;\left(X_{i}^{k}\;;\theta_{k}\right)$ using a neural network. Therefore, PIN incorporates *K* number of neural networks (Figure 2). In contrast to the regular fully connected neural network, each neural network in PIN implements a one‐to‐one variable‐selection layer between the input and the first hidden layers. Important variables are picked out by posing penalties on weights between the input layer nodes and the variable‐selection layer nodes.

Take the *k*th neural network for the *k*th dataset as an example. The neural network takes all *p* variables in $X_{i}^{k}$ as its input nodes and connects them one‐to‐one to the nodes in the variable‐selection layer. In particular, we write the one‐to‐one connection weights as $\{\;w_{1}^{k},\text{…},w_{p}^{k}\}$, which are highlighted in the colored boxes in Figure 2. The nodes of the variable‐selection layer are further inputted into the first hidden layer in a fully connected fashion. Eventually, the neural network outputs a scalar value $o_{i}^{k}$ in the output node. Typically, we have $o_{i}^{k}=f^{k}\;\left(X_{i}^{k}\;;\theta_{k}\right)$, where ***θ***_*k*_ is the set of all weights in this neural network.

The loss function of PIN is expressed as follows:

(1) $$Q\left(\theta ,\lambda ,\alpha \right)=\sum_{k=1}^{K}\sum_{i=1}^{N_{k}}\frac{1}{2N_{k}}\{Y_{i}^{k}-f^{k}{\left(X_{i}^{k};\theta_{k}\right)\}}^{2}+\sum_{j=1}^{p}P_{\lambda }\left(w_{j}\right)+\alpha {\left\|\eta \right\|}_{2}^{2}$$

where ***θ*** is the set of all parameters, $w_{j}=\left\{w_{j}^{1},\text{⋯},w_{j}^{K},j=1,\text{⋯},p\right\}$ represents the variable‐selection weights for the *j*th variable in all *K* neural networks, and ***η*** denotes the rest parameters in ***θ*** excluding ***w***_*j*_’s. PIN achieves variable selection among multiple datasets by treating each ***w***_*j*_ as a group and posing penalties *P*_*λ*_ to the weight groups. An *L*2 penalty is applied to the rest parameters ***η***, to avoid over‐fitting. As seen in Equation (1), PIN assumes that multiple datasets share the same input variables but with different values, which is reasonable in real applications. Next, we will introduce the homogeneity and heterogeneity PINs under the framework in Equation (1).

#### 5.2.2 Homogeneity and heterogeneity PIN

The homogeneity PIN (HoPIN) method addresses the homogeneity situation where the important variables are generally consistent among datasets. The network topology of HoPIN is shown in Figure 2A. We impose a group‐lasso penalty [38] on each weight group ***w***_*j*_, resulting in the selection or non‐selection of variable *j* in all datasets. The loss function of HoPIN is expressed as:

(2) $$Q\left(\theta ,\lambda ,\alpha \right)=\sum_{k=1}^{K}\sum_{i=1}^{N_{k}}\frac{1}{2N_{k}}\{Y_{i}^{k}-f^{k}{\left(X_{i}^{k};\theta_{k}\right)\}}^{2}+\sum_{j=1}^{p}\lambda {\left\|w_{j}\right\|}_{2}+\alpha {\left\|\eta \right\|}_{2}^{2}$$

The heterogeneity PIN (HePIN) method deals with the heterogeneity situation where the multiple datasets have overlapping but different sets of important variables. We impose the sparse group‐lasso penalty [39] on each weight group ***w***_*j*_, allowing the selection of variable *j* in some but not all datasets. The loss function of the HePIN is:

(3) $$Q\left(\theta ,\lambda ,\alpha \right)\;=\sum_{k=1}^{K}\sum_{i=1}^{N_{k}}\frac{1}{2N_{k}}\{Y_{i}^{k}-f^{k}{\left(X_{i}^{k};\theta_{k}\right)\}}^{2}+\sum_{j=1}^{p}\lambda_{1}\left(1-\lambda_{2}\right){\left\|w_{j}\right\|}_{2}+\sum_{j=1}^{p}\sum_{k=1}^{K}\lambda_{1}\lambda_{2}\left|w_{j}^{k}\right|+\alpha {\left\|\eta \right\|}_{2}^{2}$$

#### 5.2.3 Smoothing, optimization, and hyperparameters

The regularization terms ${\left\|w_{j}\right\|}_{2}$ and $\left|w_{j}^{k}\right|$ in loss functions (2) and (3) are non‐differentiable at the origin, which brings challenges in the optimization of the loss functions. We employ the smoothing approximation technique to address this issue [9]. For example, when $\sqrt{\sum_{k=1}^{K}{\left(w_{j}^{k}\right)}^{2}}\leq a$, we approximate ${\left\|w_{j}\right\|}_{2}$ with $S\left({\left\|w_{j}\right\|}_{2}\right)=-\{{\sum_{k=1}^{K}{\left(w_{j}^{k}\right)}^{2}\}}^{2}/8a^{3}+3\sum_{k=1}^{K}{\left(w_{j}^{k}\right)}^{2}/4a+3a/8$, here *a* is a prespecified small number. We use the default *a* = 0.04 by following Zhang et al.’s [9] suggestion. We use the gradient descent algorithm with momentum [40] to optimize the loss functions. In addition, the proposed models involve the selection of hyperparameters, including the number of hidden layers, nodes per hidden layer, learning rate, and regularization parameters. We perform 5‐fold cross‐validations to select the optimal hyperparameter set in the training data. We implement the proposed methods and the optimization algorithm in R and Python.

#### 5.2.4 Generation of simulated data

We simulate three datasets (*k* = 1, 2, 3), containing 200, 175, and 150 subjects, respectively. Each dataset contains 100 or 500 variables. The variable values are generated from the multivariate Gaussian distribution with a mean of zero and the correlation coefficients between variables *i* and *j* being 0.5^|*i* − *j*|^. For the same variable, the directions and magnitudes of its coefficients vary across different datasets, as shown in detail below.

For the homogeneity data generation, we set the first five variables associated with the outcome variable in all three simulated datasets. Moreover, we consider both linear and nonlinear variable structures. Under the linear setting, we set $Y_{i}^{k}=\beta_{1}^{k}X_{i1}^{k}+\beta_{2}^{k}X_{i2}^{k}+\beta_{3}^{k}X_{i3}^{k}+\beta_{4}^{k}X_{i4}^{k}+\beta_{5}^{k}X_{i5}^{k}+\epsilon_{i}^{k}$, where $\left(\beta_{1}^{k},\beta_{2}^{k},\beta_{3}^{k},\beta_{4}^{k},\beta_{5}^{k}\right)=\left(4,4,-8,-4,2\right),\left(-2,-2,4,-2,1\right),\left(-1.5,-1.5,3,-1.5,-0.75\right)$ for *k* = 1, 2, 3, respectively. Under the nonlinear setting, we have $Y_{i}^{k}=\beta_{1}^{k}X_{i1}^{k}+\beta_{2}^{k}X_{i2}^{k}+\beta_{3}^{k}\;\sin\left(4\pi X_{i3}^{k}\right)+\beta_{4}^{k}\;\sin\left(4\pi X_{i4}^{k}\right)+\beta_{5}^{k}\;\exp\left(X_{i5}^{k}\right)+\epsilon_{i}^{k},$ with the coefficients $\left(\beta_{1}^{k},\beta_{2}^{k},\beta_{3}^{k},\beta_{4}^{k},\beta_{5}^{k}\right)=\left(4,4,-8,-8,2\right),\left(-2,-2,4,-4,1\right)$, and (−1.5, −1.5, 3, −3, −0.75) for *k* = 1, 2, 3, respectively. The error terms $\epsilon_{i}^{k}$ are generated from the Gaussian distribution.

For the heterogeneity data generation, we define the three simulated datasets to have different sets of important variables. Under the linear setting, we have $Y_{i}^{k}=\beta_{1}^{k}X_{i1}^{k}+\beta_{2}^{k}X_{i2}^{k}+\text{…}+\beta_{10}^{k}X_{i10}^{k}+\epsilon_{i}^{k}$, with $\left(\beta_{1}^{k},\beta_{2}^{k},\text{…},\beta_{10}^{k}\right)=\left(4,4,-8,-4,2,-6,-3,0,0,0\right),\left(-2,-2,4,-2,1,0,0,3,-1,0\right)$, and (−1.5, −1.5, 3, −1.5, −0.75, 0, 0, 0, 0, 2) for *k* = 1, 2, 3, respectively. In the nonlinear setting, we have $Y_{i}^{k}=\beta_{1}^{k}X_{i1}^{k}+\beta_{2}^{k}X_{i2}^{k}+\beta_{3}^{k}\;\sin\left(4\pi X_{i3}^{k}\right)+\beta_{4}^{k}\;\sin\left(4\pi X_{i4}^{k}\right)+\beta_{5}^{k}\;\exp\left(2.5X_{i5}^{k}\right)+\beta_{6}^{k}\;\exp\left(2.5X_{i6}^{k}\right)+\beta_{7}^{k}\;\exp\left(2.5X_{i7}^{k}\right)+\beta_{8}^{k}\;\exp\left(2.5X_{i8}^{k}\right)+\epsilon_{i}^{k}$, with $\left(\beta_{1}^{k},\beta_{2}^{k},\text{…},\beta_{8}^{k}\right)=\left(4,4,-8,-8,2,-2,0,0\right),\left(-2,-2,4,-4,1,0,1,0\right)$, and (−1.5, −1.5, 3, −3, −0.75, 0, 0, 0.75) for *k* = 1, 2, 3, respectively. The error terms $\epsilon_{i}^{k}$ are generated from the Gaussian distribution.

## AUTHOR CONTRIBUTIONS

**Yang Li**: Project administration; conceptualization; supervision; funding acquisition; writing –review & editing. **Xiaonan Ren**: Data curation; formal analysis; methodology; software; validation; visualization; writing – original draft, review & editing. **Haochen Yu**: Data curation; formal analysis; methodology; software. **Tao Sun**: Data curation; project administration; supervision; funding acquisition; writing –review & editing. **Shuangge Ma**: Writing – review & editing.

## CONFLICT OF INTEREST STATEMENT

Yang Li, Xiaonan Ren, Haochen Yu, Tao Sun and Shuangge Ma declare that there exists no conflict of interest or financial impropriety that necessitates disclosure.

## ETHICS STATEMENT

All procedures performed in studies were in accordance with the ethical standards of the institution or practice at which the studies were conducted, and with the 1964 Helsinki declaration and its later amendments or comparable ethical standards.

## Supporting information

Supporting Information S1

## Data Availability

The datasets used during the analysis of Alzheimer's disease are available in the Alzheimer's Disease Neuroimaging Initiative study. The datasets used during the analysis of ovarian cancer are available in the Bioconductor R package “CuratedOvarianData”.
