# Supplementary material for: A penalized integrative deep neural network for variable selection among multiple omics datasets
Source: Quant Biol. 2024 Jun 7;12(3):313–23. doi: 10.1002/qub2.51 (PMC12806554; doi:10.1002/qub2.51)
Supplement: Supplementary file 1 — Supporting Information S1 [file QUB2-12-313-s001.pdf]

A penalized integrative deep neural network for variable selection among multiple omics datasets

Supporting File

Yang Li[1],Xiaonan Ren[1],Haochen Yu[1],Tao Sun[1*],Shuangge Ma[2]

[1] Center for Applied Statistics, School of Statistics, Renmin University of China, Beijing, 100872, China

[2] Department of Biostatistics, Yale University, New Haven, CT 06511, United States

[*] sun.tao@ruc.edu.cn

May 31, 2024

1 Additional real data analysis results

Table S1: List of important genes and their corresponding weight values selected by HoPIN in the Alzheimer's disease datasets.

Gene	CN	MCI	AD
SAV1	0.0073	0.0119	0.0732
PLBD2	0.0119	0.0062	0.0403
LZTS1	0.0094	0.0189	0.0500
EIF4EBP1	-0.0067	0.0057	0.0418
IL36A	0.0077	0.0104	0.0389
PMM2	0.0195	0.0175	0.0477
TAF4B	0.0012	0.0040	0.0079
SNAI3	0.0135	0.0154	0.0806
SLC45A1	0.0074	0.0102	0.0821
NEUROD1	0.0335	0.0159	0.0669
CUEDC1	0.0378	0.0095	0.0454
SPDYE6	0.0005	0.0116	0.0624
LHX2	0.0099	0.0174	0.0434
GPSM2	0.0094	0.0098	0.0771
DOCK10	0.0125	0.0072	0.0632
FOXI1	0.0198	0.0039	0.0626
SLAIN1	0.0092	0.0096	0.0450
RAD23B	0.0079	0.0082	0.0440
EPB41L2	0.0274	0.0096	0.0599
IL20RB	0.0079	0.0033	0.0621
CPT2	0.0080	0.0074	0.0675
ACOX3	0.0075	0.0074	0.0420

Table S2: List of important genes and their corresponding weight values ($\times 100$) selected by HePIN in the ovarian cancer datasets.

Gene	TCGA	GSE26712	GSE32062	GSE9891
NAB1		0.278		0.179
RBPJL	0.269	0.208		
NT5C		0.449		
GALNT10	0.167			
HSD17B6		0.520		
LRIG1	0.085			
GFPT2		0.243		
PSEN2		-0.205		0.225
RHOBTB3		0.310		0.327
WDR77	0.200			
CLPS	0.120	0.231		
HIPK1		-0.272		
CALB2	0.080			
XYLT1			-0.135	
ZFHX4		0.218		
WISP1		-0.265		
TIMP3			0.148	
SH2B3		0.535		
SLC37A4	0.094			
ACY1				0.158
FGF1				0.169
SRSF8	0.276			
GJB1				0.173
CD302	-0.113	-0.238		
ARNT	0.184			
BICC1		-0.366		-0.197
CALML3		0.252		
FAM69A			0.189	
MORN1		0.478		0.223
BLMH		0.248		
NUAK1		0.319		
OXLD1		0.270		
FUZ				0.189
NBL1		-0.318		
PPP1R14B		-0.290		
CHST15		0.368		
SNAI2		0.286		
TOPORS	0.079			
EDNRA		0.270		
HSD17B8		-0.236		
CPEB1		0.273		0.129
SOAT1		-0.254		
HOXB7			0.133	
PARP3			0.173	
FILIP1L		0.461		
NDUFA7		0.294		0.161
DOK5				0.335
PAQR4		-0.235		
HCN2				0.210
ZNHIT2		-0.238		
OSR2			-0.174	
CETN2		0.243		
CSGALNACT2		-0.346		0.146
IGFBP6		0.269		
DCP2				0.256
AKT3		0.395		
KCNG2	0.094			
RARRES1		0.446		
LYPD1				0.133
ORAI2				0.208
CHL1			0.190	
KIAA1033		0.286		
SLCO2B1		0.252		
ALB	0.110			
PEX26				0.206
PJA2		-0.207		
CILP			0.284	
MTX2		0.273		
GSTZ1		0.265		
ACTA2		0.301		
MATN4		0.292		
FARS2		-0.275		
KIAA1199			0.170	
ADPRM	0.101			
C5AR1		-0.299		
ZMAT3		-0.210		
PIK3R1				0.171
CALD1			-0.134	
KCNF1				0.146
MAN2A1		0.236		
IGFBP4			-0.137	
MVD	-0.086			
ACOT1			0.135	
COL3A1		0.239		
CLDN10	0.112			
LGALS14		0.322		
CMTM6		0.228	0.132	
CCL24			0.191	0.177
EHD2		0.220		
SPCS2	0.131			
PSME1		0.246		
ZEB1		0.260		
LPAR2		0.373		
SFRP5	0.099	0.251		0.305
TREML2	0.197	-0.238		
RPS6KB2		0.362		
SERPINF1		0.295		
HIST1H2BJ				0.207

Gene	TCGA	GSE26712	GSE32062	GSE9891
UEVLD			0.207	
TLE6		-0.272		
MICAL2	0.084	0.623		
PUF60		0.483		
GLRX		0.229		
HOXA5		-0.275		
TTBK2				0.335
KIAA1462	0.110			
KCNQ4				-0.176
SLC2A5		0.256		
TGFB1		0.218		
ZEB2	0.108			
ZNF16		0.666		
TSGA10				0.205
PACSIN3		0.549		
TGFB1I1			0.152	
TRMU		0.433		
HNRNPC			0.140	
PICK1			-0.134	
AXL		-0.224		
HYAL2		0.421		
NFX1		0.226		
CCNH				-0.172
PDPN		0.204		
SLC7A11	0.093			
TELO2			0.139	
PIGZ				0.201
SLC25A10		-0.238		0.148
NID2			-0.157	
SLC5A3		0.258		
PTGFR	0.173			
COTL1				0.182
SPON2		-0.207		
NPPB		0.292		
NUDT3			0.142	
MIF			0.172	0.170
PTGES2				0.135
C8A				0.128
ITGA5		-0.235		
FAAH				0.250
EYA2			0.160	
NFS1	1.537	0.931	2.631	2.540
CCDC40				0.247
EFEMP1	0.084			
WBP1L	-0.095			0.237
GIT2				0.140
ENPP1				0.148
DNASE1	0.276			
HOXA9	0.090			
SMARCD2		0.237		
APOBEC3F		0.463		
ALDH1A3		-0.208		
VAT1			0.179	
AOC3		-0.228		
ID3		0.240	0.135	
PDHB	1.206	4.043	1.073	1.203
TLX2		0.242		
UCP3	0.098			
HOXD3		0.381		
APOC3	-0.082	0.243		
USP8	0.080	0.400		
NR2E3		-0.272		
CD93		0.225	0.214	
FAT2	0.084		0.287	
PRSS50		0.281		
NARF		0.264		
GTF2H4		0.355		
NBEAL2		0.252		
MMP2		-0.263		
PMS1	0.095			
NR1D1			0.203	
TAOK3		0.226		
HTR2B				0.130
SCGB1A1			0.147	
KIF26B				-0.209
ZNF76		-0.233		
TGM2		0.248		
SCARB2		0.218		
HEXB	0.103			
ELMO2	0.088			
PFKFB4			0.140	
TIMM8B		-0.219		
PIN1P1		0.285		
B4GALT5	0.169	0.469		0.174
RNF186	0.284			
TUBB4A	0.101			0.218
PCK2		0.370		
TSKS		-0.293		
PDGFD		0.268		
ZCCHC14				0.205
UVRAG		0.238		
TESK2	0.161			
PCDHB11		0.317		
SERPINA4	0.108			
ST6GALNAC4			-0.176	
GKN1	0.868	3.687	0.931	0.981
TGFB1	0.099			

2 Additional simulations

2.1 $p = 500$

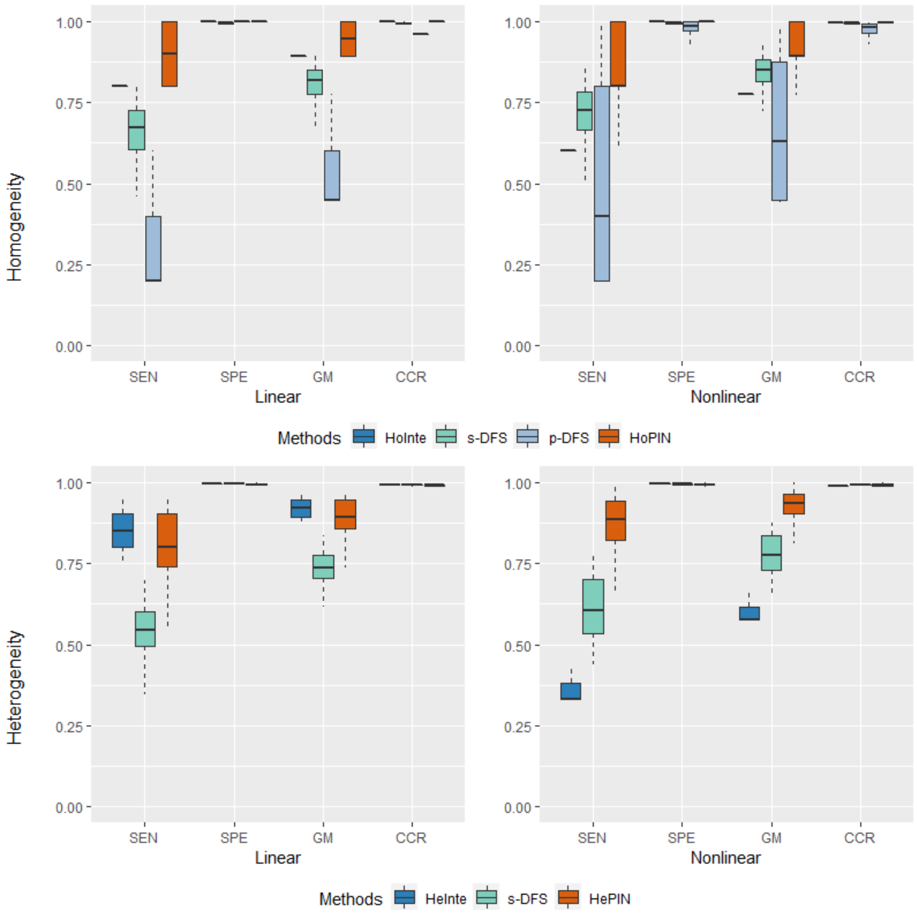

Figure S1: Boxplots of variable selection metrics (SEN, SPE, GM, and CCR) under the homogeneity (upper) and heterogeneity (below) situations where the variable structure is linear (left) or non-linear (right). Three datasets are generated with a total of 525 subjects and 500 variables. Fifty replications of simulations are performed. SEN stands for sensitivity, SPE for specificity, GM for the geometric mean of sensitivity and specificity, and CCR for correct classification rate. Higher values of the four metrics indicate better selection performance.

Table S3: The mean squared error averages and standard deviations under the homogeneity (I) and heterogeneity (II) situations when the variable structure is linear or nonlinear. The number of predictors is set at $p = 500$.

		Linear	Nonlinear
(I) Homogeneity	HoPIN	0.015(0.004)	0.023(0.005)
	HoInte	0.011(0.002)	0.023(0.005)
	p-DFS	0.016(0.005)	0.022(0.006)
	s-DFS	0.017(0.005)	0.025(0.005)
(II) Heterogeneity	HePIN	0.016(0.003)	0.022(0.004)
	HeInte	0.014(0.003)	0.029(0.005)
	s-DFS	0.020(0.004)	0.026(0.005)

2.2 Scenario under there no overlap of important variables among all datasets.

We simulate three datasets, containing 200, 175, and 150 subjects, respectively. Each dataset contains 100 variables. The variable values are generated from the multivariate Gaussian distribution with a mean of zero and the correlation coefficients between variables i and j being $0.3^{|i-j|}$. There is no overlap of important variables among three datasets, as shown in detail below:

$$\text{dataset1: } Y_i^1 = 10X_{i1}^1 + 7X_{i2}^1 - 15e^{1.5X_{i3}^1} + \varepsilon_i^1, i = 1, 2, \dots, 200$$
$$\text{dataset2: } Y_i^2 = -3e^{X_{i4}^2} + e^{2.5X_{i5}^2} + 2X_{i6}^2 + \varepsilon_i^2, i = 1, 2, \dots, 175$$
$$\text{dataset3: } Y_i^3 = -10.5X_{i7}^3 + 3e^{3X_{i8}^3} - 3e^{2.5X_{i9}^3} + \varepsilon_i^3, i = 1, 2, \dots, 150$$

where the error terms $\varepsilon_i^k (k = 1, 2, 3)$ are generated from the Gaussian distribution. We evaluate three methods: s-DFS, HoPIN, and HePIN. Considering the model setting is a nonlinear structure, we did not use Inte as a comparison. We randomly select 80% subjects as training data and the rest as test data. We repeat this process 50 times. Sensitivity (SEN), specificity (SPE), the geometric mean of sensitivity and specificity (GM), the correct classification rate (CCR), and the mean of squared errors (MSE) are be used to evaluate model performance.

The variable selection performance is illustrated in Figure S3. s-DFS generally achieves the most optimal variable selection performance based on all four metrics, as it conducts variable selection separately for each dataset and effectively identifies important variables. HePIN also demonstrates a good variable selection performance. Considering that we were not aware of the absence of overlap of important variables between the datasets in advance, we did not include 0 as an option for the penalty term during the selection of hyperparameters, which may impact the accuracy of selection. HoPIN exhibits the lowest performance because it cannot handle the situation that there is no overlap of important variables among all datasets. In fact, HoPIN consistently selected variables 1-9 along with some other unimportant variables, which resulting in high SEN and low SPE. Therefore, our proposed method HePIN can also perform well when there is no overlap of important variables among all datasets, which is comparable to performing DFS separately for each dataset.

For prediction performance, we only compare the results of HePIN and s-DFS here. The mean squared error averages and standard deviations under HePIN is 0.0164 (0.003), and under s-DFS is 0.0199 (0.009). HePIN achieves better prediction performance due to the larger sample size.

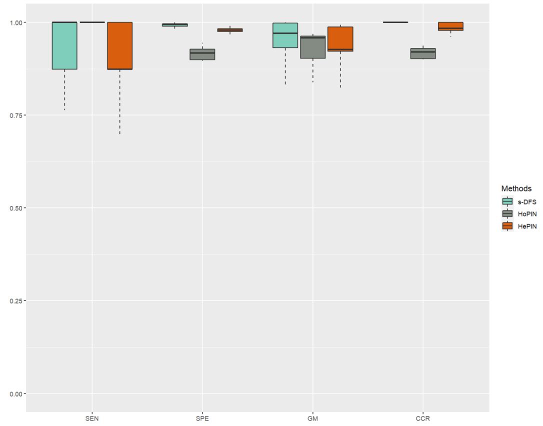

Figure S2: Boxplots of variable selection metrics (SEN, SPE, GM, and CCR) under the scenario of no overlapping important variables. Three datasets are generated with a total of 525 subjects and 100 variables. Fifty replications of simulations are performed. SEN stands for sensitivity, SPE for specificity, GM for the geometric mean of sensitivity and specificity, and CCR for correct classification rate. Higher values of the four metrics indicate better selection performance.

3 HePIN under homogeneity data

We adopt HePIN for variable selection under the assumption of homogeneity data and compare it with HoPIN. Suppose there are three datasets ($k = 1, 2, 3$), each containing 200, 175, or 150 subjects. Each dataset con-

tains 100 variables. The variable values are generated from the multivariate normal distribution, correlation coefficients between variables i and j being $0.3^{|i-j|}$, and we set $Y_i^k = \beta_1^k X_{i1}^k + \beta_2^k X_{i2}^k + \beta_3^k \sin\left(4\pi X_{i3}^k\right) + \beta_4^k \sin\left(4\pi X_{i4}^k\right) + \beta_5^k \exp\left(2.5 X_{i5}^k\right) + \varepsilon_i^k$, where $\left(\beta_1^k, \beta_2^k, \beta_3^k, \beta_4^k, \beta_5^k\right) = (4, 4, -8, -8, 2), (-2, -2, -4, -4, 1)$, and $(-1.5, -1.5, 3, -3, -0.75)$ for $k = 1, 2, 3$. The variable selection performance is illustrated in Figure **??**. HePIN's performance is similar to that of HoPIN, indicating that HePIN can also identify important variables under homogeneous scenarios.

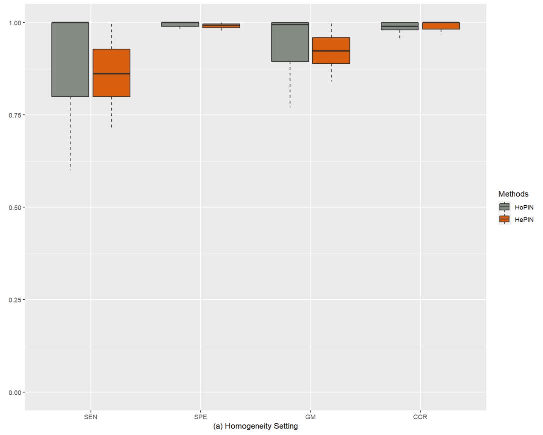

Figure S3: Boxplots of variable selection metrics (SEN, SPE, GM, and CCR) under the homogeneity data. Three datasets are generated with a total of 525 subjects and 100 variables. Fifty replications of simulations are performed. SEN stands for sensitivity, SPE for specificity, GM for the geometric mean of sensitivity and specificity, and CCR for correct classification rate. Higher values of the four metrics indicate better selection performance.

4 Two toy examples

4.1 A toy example for HoPIN

Here, we present a toy example for applying HoPIN using simulated data.

4.1.1 Data generation under the homogeneity situation

Suppose there are three datasets ($k = 1, 2, 3$), each containing 200, 175, or 150 subjects. Each dataset contains 50 variables. The variable values are generated from the multivariate normal distribution, with correlation coefficients between variables i and j as $0.5^{|i-j|}$. We assume that the same five variables are associated with the outcomes in the three datasets and generate data in the following settings:

$$
\begin{aligned}
Y_i^1 &= 4X_{i1}^1 + 4X_{i2}^1 - 8\sin(4\pi X_{i3}^1) - 8\sin(4\pi X_{i4}^1) + 2\exp(X_{i5}^1) + \epsilon_i^1, \\
Y_i^2 &= -2X_{i1}^2 - 2X_{i2}^2 - 4\sin(4\pi X_{i3}^2) - 4\sin(4\pi X_{i4}^2) + \exp(X_{i5}^2) + \epsilon_i^2, \\
Y_i^3 &= -1.5X_{i1}^3 - 1.5X_{i2}^3 + 3\sin(4\pi X_{i3}^3) - 3\sin(4\pi X_{i4}^3) - 0.75\exp(X_{i5}^3) + \epsilon_i^3,
\end{aligned}
$$

where ${Y_i}^k$ denotes the continuous response variable and $\boldsymbol{X}_i^k = (X_{i1}^k, X_{i2}^k, \cdots, X_{ip}^k)'$ denotes the p-dimensional variable for the i_{th} subject in the k_{th} dataset. The measurement errors ϵ_i^k are generated from Gaussian errors. The heatmap of variable effects in Figure S4(a) demonstrates the homogeneity situation for the simulated data.

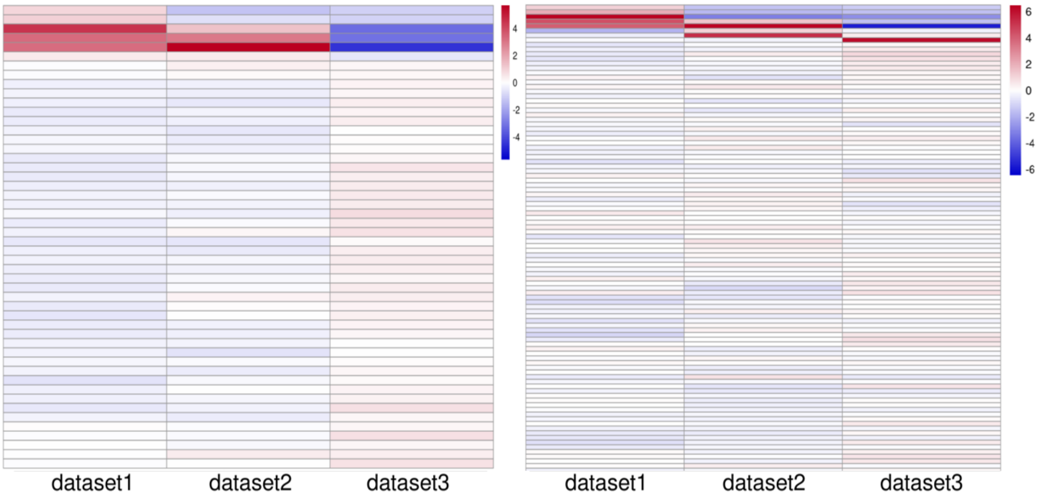

(a) HoPIN (b) HePIN

Figure S4: The heatmaps of variable effects under the homogeneity (a) and heterogeneity (b) situations. The columns represent the different datasets, and the rows indicate the variables. Positive, negative, and magnitude of variable effects are shown by red, blue, and the shade of the color.

4.1.2 Data preprocessing and hyperparameter selection

We apply normalization for all variables in each dataset. We also scale the response values to lie between $[0, 1]$. These data preprocessing procedures improve PIN's computational speed and stability. Since HoPIN involves hyperparameters and penalty parameters, we use 5-fold cross-validations to select optimal parameters based on the prediction performance. For this toy example, we select one hidden layer, $\sqrt{p}$ nodes per layer, learning rate $\eta = 0.3$, variable selection penalty parameter $\lambda = 0.02$, and hidden layer penalty parameter $\Lambda = 0.00001$.

4.1.3 Variable selection using selected hyperparameters

We use the selected parameters to train HoPIN in the training dataset. We select the important variables following Li et al. [1]. We use the criteria of $\bar{\boldsymbol{w}}_p \geq \theta \cdot \|\bar{\boldsymbol{w}}\|_\infty$, where $\bar{\boldsymbol{w}}_p$ is the average variable-selection layer weight corresponding to the p-th variable of three datasets, $\bar{\boldsymbol{w}}$ is the average variable-selection layer weights vector of three datasets, and θ is a pre-defined value 0.15. Figure S5 illustrates the estimated variable-selection layer weights for all 50 variables in each dataset.

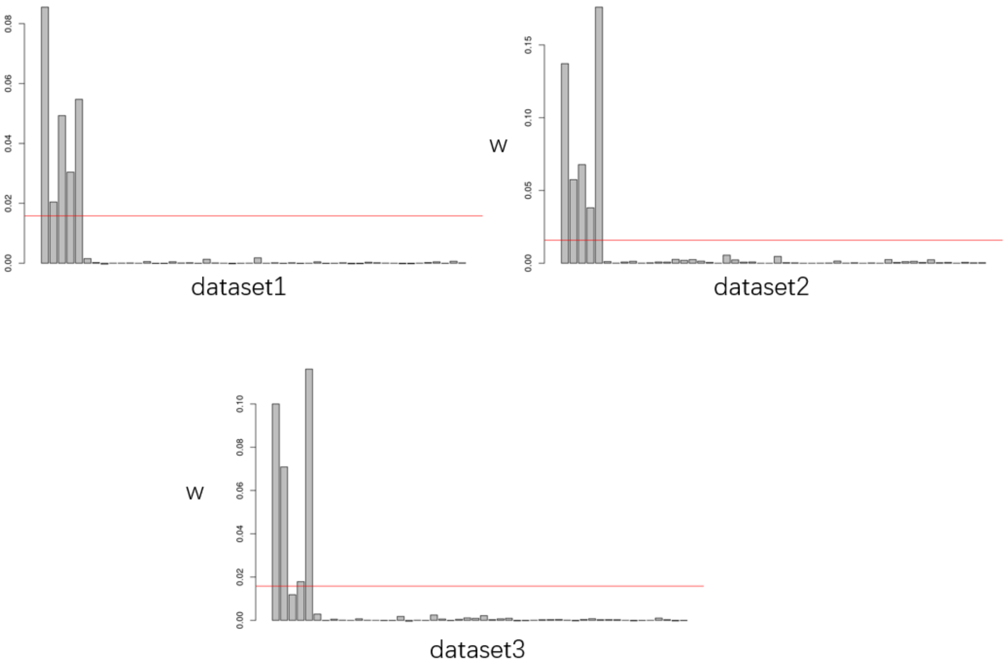

Figure S5: The variable-selection layer weights from HoPIN in the three datasets of the toy example. The y-axis indicates weight values, and the x-axis indicates variable indexes. The red line indicates the variable selection threshold.

4.2 A toy example for HePIN

We present a toy example for applying HePIN using simulated data.

4.2.1 Data generation under the heterogeneity situation

Suppose we have three datasets ($k = 1, 2, 3$), each containing 200, 175, or 150 subjects. Each dataset has 100 variables. The variable values are generated from the multivariate normal distribution, with correlation coefficients between variables i and j as $0.3^{|i-j|}$. We assume that the important variables associated with the outcomes in the three datasets are overlapping but not the same. Assign the important variables in each dataset as: dataset 1: 1, 2, 3, 4, 5, 6; dataset 2: 1, 2, 3, 4, 5, 7; dataset 3: 1, 2, 3, 4, 5, 8. We generate data in the following setting:

$$
\begin{aligned}
Y_i^1 &= 4X_{i1}^1 + 4X_{i2}^1 - 8\sin(4\pi X_{i3}^1) - 8\sin(4\pi X_{i4}^1) + 2\exp(2.5X_{i5}^1) - 2\exp(2.5X_{i6}^1) + \epsilon_i^1,\\
Y_i^2 &= -2X_{i1}^2 - 2X_{i2}^2 + 4\sin(4\pi X_{i3}^2) - 4\sin(4\pi X_{i4}^2) + \exp(2.5X_{i5}^2) + \exp(2.5X_{i7}^1) + \epsilon_i^2,\\
Y_i^3 &= -1.5X_{i1}^3 - 1.5X_{i2}^3 + 3\sin(4\pi X_{i3}^3) - 3\sin(4\pi X_{i4}^3) - 0.75\exp(2.5X_{i5}^3) + 0.75\exp(2.5X_{i8}^1) + \epsilon_i^3,
\end{aligned}
$$

where Y_i^k denotes the continuous response variable and $\boldsymbol{X}_i^k = (X_{i1}^k, X_{i2}^k, \cdots, X_{ip}^k)'$ denotes the p-dimensional variable for the i_{th} subject in the k_{th} dataset. The measurement errors ϵ_i^k are generated from Gaussian errors. The heatmap of variable effects in Figure S4(b) illustrates the heterogeneity situation for the simulated datasets.

4.2.2 Data preprocessing and hyperparameter selection

In this example, the selected parameters are: one hidden layer, $\sqrt{p}$ nodes per layer, $\eta = 0.3, \lambda_1 = 0.02, \lambda_2 = 0.05$, and $\Lambda = 0.00001$.

4.2.3 Variable selection using selected hyperparameters

We use the selected parameters to train HePIN in the training dataset. Figure S6 illustrates the estimated variable-selection layer weights for all 100 variables in each dataset. We select the important variables following Li et al. [1] by using the criteria of $w_p^k \geq \theta \cdot \|\boldsymbol{w}^k\|_\infty$, where w_p^k is the variable-selection layer weight corresponding

to the p-th variable in dataset k, $\boldsymbol{w}^k$ is the variable-selection layer weights vector of dataset k, and θ is a pre-defined value 0.15. The selected important variables are those variables above the red lines as illustrated in Figure S6.

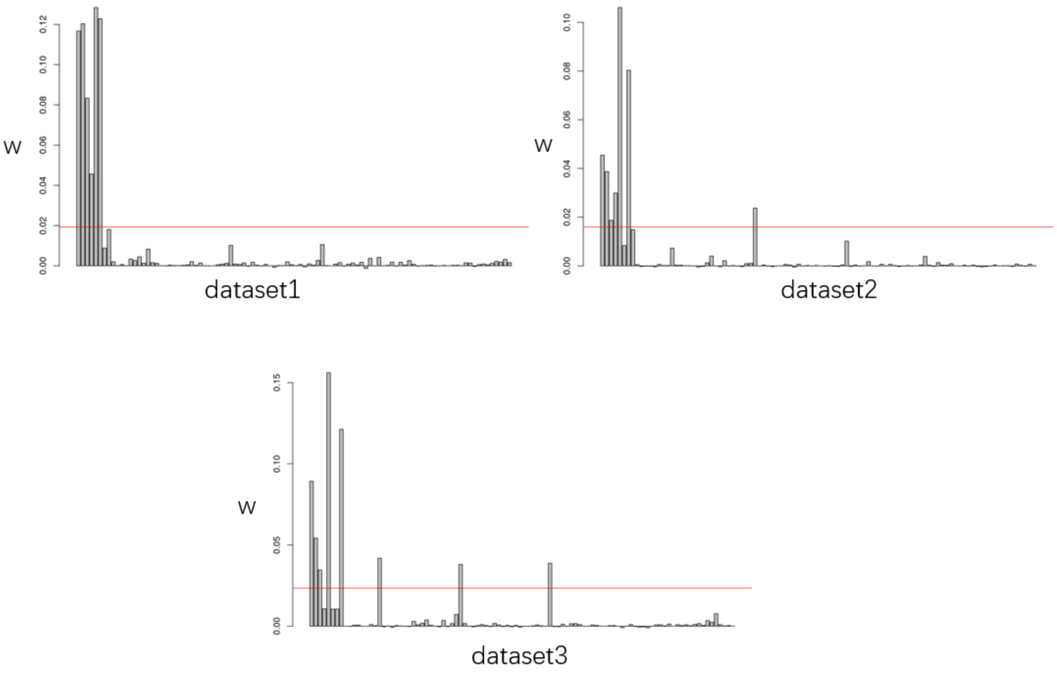

Figure S6: The variable-selection layer weights from HePIN in the three datasets of the toy example. The red line indicates the variable selection threshold.

References

[1] Y. Li, C.-Y. Chen, and W. W. Wasserman, "Deep feature selection: theory and application to identify enhancers and promoters," *Journal of Computational Biology*, vol. 23, no. 5, pp. 322–336, 2016.
